# Reply to: Evidence confirms an anthropic origin of Amazonian Dark Earths

**DOI:** 10.1038/s41467-022-31065-1

**Published:** 2022-06-17

**Authors:** Lucas C. R. Silva, Rodrigo Studart Corrêa, Jamie L. Wright, Barbara Bomfim, Lauren B. Hendricks, Daniel G. Gavin, Aleksander Westphal Muniz, Gilvan Coimbra Martins, Antônio Carlos Vargas Motta, Julierme Zimmer Barbosa, Vander de Freitas Melo, Scott D. Young, Martin R. Broadley, Roberto Ventura Santos

**Affiliations:** 1 Environmental Studies Program, University of Oregon, Eugene, OR USA; 2 Department of Geography, University of Oregon, Eugene, OR USA; 3 Environmental Sciences Program - PPGCA/FUP, University of Brasília, Planaltina, DF Brazil; 4 Climate and Ecosystem Sciences Division, Lawrence Berkeley National Laboratory, Berkeley, CA USA; 5 Brazilian Agricultural Research Corporation - CPAA/Embrapa Amazônia Ocidental, Manaus, AM Brazil; 6 Department of Soil Science, University of Paraná, Curitiba, PR Brazil; 7 Federal Institute of Southeast Minas Gerais, Barbacena, Minas Gerais Brazil; 8 School of Biosciences, University of Nottingham, Nottingham, UK; 9 Institute of Geosciences, University of Brasília, Brasilia, DF Brazil

**Keywords:** Element cycles, Palaeoecology, Limnology

**replying to** Lombardo et al. *Nature Communications* 10.1038/s41467-022-31064-2 (2022)

Amazonian Dark Earths (ADEs) are widely regarded as a model for sustainable agriculture. Their unusual fertility and elevated concentration of charcoal, combined with the frequent occurrence of pre-Columbian artifacts at ADE sites, has prompted widespread biomass burning for soil amendment in tropical regions. However, it remains unclear how these persistent patches of high fertility could have been created in nutrient-impoverished tropical landscapes. In a recent study^1^, we report new data from one of the best-studied ADE sites in Brazil which warrant a revision of its origin story. We found large amounts of phosphorus (P) and calcium (Ca) correlated with 16 trace elements that indicate exogenous sources rather than in situ deposition, an inference that is supported by neodymium (Ne), strontium (Sr) and carbon (C) isotope signatures. Moreover, radiocarbon (^14^C) activity of charcoal in ADEs suggested inputs beginning thousands of years before the earliest evidence of forest clearing for agriculture in the region. Our results imply that indigenous populations either managed soils at the site thousands of years earlier than previously reported or, alternatively, that human-derived inputs represent a small fraction of ADE’s chemical makeup, a fraction that, we hypothesise based on the size and timing of deposition (Fig. 1), was introduced in the relatively recent past.

**Fig. 1:**
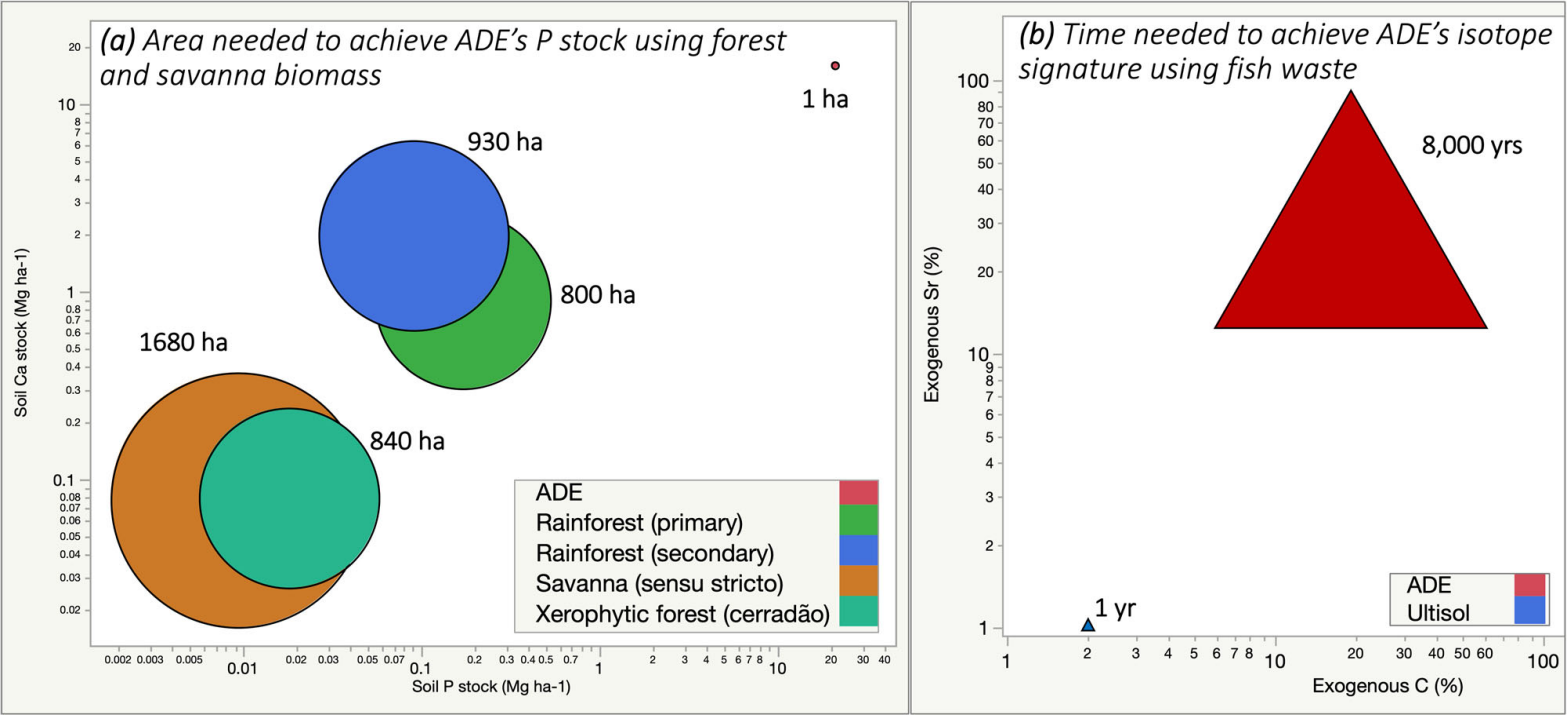
Nutrient stocks needed to form Amazonian Dark Earths (ADEs). **a** The coordinates of the points show the measured P and Ca stocks in the top 1 m of soil (log-scale axis) in ADEs^1^ and in above-ground biomass of tropical forests and savannas^20–23^. The size of circles: (not logged) represents the area needed to concentrate P from the biomass of each ecosystem into a 1-hectare ADE patch (upper-right point) to achieve the observed fertility. The areas are calculated as the quotient of the P stock in 1 ha of ADE soil and the P concentration in above-ground biomass (e.g., for primary rainforest). These estimates combine wood and foliage biomass measured in contemporary landscapes^20–23^ conservatively assuming no elemental loss after biomass deposition or burning. **b** The coordinates of the points show the estimated amount of organic C and Sr mass in the top 1 m of soil (log-scale axis), originating from exogenous inputs based on isotopic signatures as presented in Silva et al.^1^ For Sr, radiogenic signatures indicate that ~24% of Sr mass in ADE profiles originate from either fishbone or river sediment as both sources have the same Sr isotopic composition. For C, grass-derived biomass from savannas upstream could explain the change in C source through the profile. The size of triangle: (not logged) represents time of occupation needed to achieve ADE nutrient stocks from fish biomass assuming no elemental losses during 8000 years of continued occupation (20 people ha^−1^ consuming 90 g of fresh fish day^−1^).

Lombardo et al.^2^ disagree and dispute our interpretation. They raise important questions which were addressed in our original paper albeit not in detail. Here, we expand on our previous analysis to estimate the land area and time needed to explain ADE formation through human inputs. This new analysis offers further support to our conclusion that indigenous peoples harnessed natural processes of elemental deposition, which led to the unique properties of ADEs, underscoring the need for a broader view of landscape evolution to redirect sustainable land use in the region. As explained in our study^1^, evidence from a single (albeit iconic) ADE site should not be extrapolated across the entire basin. However, our findings do raise general questions about previously proposed timing and mechanisms of ADE formation. We argue that our hypothesis should be tested in other sites through interdisciplinary research that combines indigenous knowledge, neotectonics, fluvial geomorphology, and a modern understanding of elemental cycling. Such an approach could uncover the mystery of ADE formation and guide the sustainable use of tropical landscapes going forward, hopefully leading to new discoveries of regional and global significance.

## Elemental stocks

Lombardo et al. argue that previous studies *“confirm an anthropic origin of ADEs”*. They suggest that the elemental concentrations observed at our site could be obtained from concentrating and burning biomass in a typical small village of pre-Columbian Amazonia. Specifically, they suggest that a 50-hectare catchment could yield enough wood debris and waste that could concentrate by combustion into a 0.1-hectare patch. However, we calculated the elemental stocks across the entire patch and obtained values which challenge this explanation. Our original paper included inputs from a range of human wastes (including faeces and fish carcasses), revealing unrealistic area or time of occupation to account for the observed elemental stock. Here, we add a second analysis for wood ash and charcoal. Considering elemental stocks measured in tropical forest or savanna biomass, the area required to attain the excess amount of phosphorus (P) at our 12-hectare sampling patch would be 9600–20,160 hectares (Fig. 1; left panel). Such an intensity of land clearance and burning would cross a disturbance threshold for tropical forests, resulting in grass and shrub vegetation not easily converted back to forest.

As explained in our original article^1^, ~5300 Mg ha^−1^ of fresh fish biomass would be needed to explain ADE’s nutrient excess, assuming 20 people per hectare each consuming 90 g of fresh fish per day. In this scenario, the formation of an ADE patch would have required ~8000 years of continued occupation (Fig. 1; right panel). If human faeces were the source of nutrients, the time necessary would be ~11,000 years^1^. These are conservative estimates that disregard nutrient losses after biomass deposition. Yet, these estimates greatly surpass the previously proposed chronology of occupation for ADE sites in central Amazonia. As nutrients are lost when biomass is removed or burned, tree cover declines and grass cover expands creating savannas within a few decades of repeated disturbance^3^. If we were to account for those losses, the total amount of biomass needed would be orders of magnitude larger than those presented here. Thus, Lombardo et al.’s estimate does not explain our observations. Therefore, we frame our approach focused on the elemental stock and isotope signatures as a new hypothesis, distinct from earlier attempts to address a geogenic origin of ADEs.

## Exogenous inputs

Lombardo et al. cite a previous study in which we interpreted differences in nutrient concentrations as evidence of anthropic origin for ADEs^4^. That study did not extrapolate the concentration data into elemental stocks for the whole patch and did not include the isotopic data, which now allow us to distinguish in situ from exogenous inputs. Under further analysis, it became clear that the elemental pool and sources at our site could not be explained by human activity. Therefore, in Silva et al.^1^, we focused on the absolute pool size of nutrients, their isotopic ratios, and what would be required to generate those values. If our new hypothesis holds true other sites will exhibit similarly large elemental pools and biogeochemical signatures as those we reported. Unfortunately, Lombardo et al. do not address the discrepancies between the current paradigm of ADE formation and our new observations.

Regarding our inference of exogenous nutrient inputs, Lombardo et al. argue that isotopic signatures of Sr and Nd can only be used to assess provenance of fresh materials. However, the use of Sr and Nd isotopes to infer nutrient sources is a well-established approach in archeology and geochemistry^5–8^. To the best of our knowledge, that approach has not been previously disputed. Moreover, Lombardo et al. conflate Sr and Nd concentrations with isotopic ratios, which leads to an erroneous interpretation of our data. For example, they use current elemental levels in river water (without isotopic data to indicate provenance) to argue that rivers are not a significant source of elements to ADEs. Evidently, the concentration of elements in the river water today is not a proxy for depositional events that occurred thousands of years ago. We know that elemental concentrations vary widely in river water and that elemental ratios of sediment deposited in soil change over time^9^. By contrast, isotopic signatures are preserved and ours are consistent with those observed in other depositional sites prior to human occupation (discussed in our paper).

Lombardo et al. offer no explanation to our finding of C isotope ratios that indicate a savanna component prior to human occupation in the ADEs but not in the adjacent soils. Such a pattern could arise by exogenous carbon sources deposited in the ADE site. Interestingly, they do suggest that the relevant age to understand ADE formation, and whether it is consistent with human occupation, is that of silt-sized charcoal. As explained in our paper, our pyrogenic carbon extraction method retains silt-sized particles. Thus, their criticism does not hold. They also mention that recent pyrogenic carbon could have been added to shallow depths of ADEs more recently. We agree with that comment, however, our dates did not differ between the ADEs and Ultisols, for both bulk and the pyrogenic carbon pools. In fact, our pyrogenic carbon dates of the silt-sized charcoal fraction (though not many available) show mid-Holocene dates within the top 40 cm of the ADE, and there was no increase in pyrogenic carbon moving upward within the ADE soil profile, as would be expected if there were large charcoal inputs in the late Holocene from human activity. Pedogenic processes likely reworked carbon into greater depths at similar rates in both soil types, which is yet another finding that is not explained by Lombardo et al.

## Regional significance

Lombardo et al. argue that, if our hypothesis is correct, ADEs should be continuous rather than patchy. However, alluvium deposition can be a patchy process and the distribution of large and small ADE patches can be predicted regionally based on fluvial geomorphology. For example, 89% of all known ADEs have been predictively mapped using elevation, distance to bluff, and geological provenance as the key predictors (with a false negative rate of 6.5% and a false positive rate of 4.7%)^10^. Predicted areas include small and large ADE patches, up to several square kilometres in size, and indicate that ADEs cover ~154,000 km^2^ mostly in central and western Amazonia. This may seem to be a very large area (>3% of the Amazon basin) but it is only a fraction of the projections found in some of the most cited anthropogenic theory literature^11^. Assuming the same excess fertility observed at our site, the creation of those ADEs would have required a prohibitive amount of biomass burning, in areas 800–1680 times larger (Fig. 1), which is inconsistent with the centralised small-scale deposition proposed by Lombardo et al. In this regional scenario, it remains unclear how many Amazons would have been needed to build the already-mapped ADEs.

Lombardo et al. centre their opinion on settlements in other parts of the Amazon basin, under different socioecological and geomorphological contexts, and where the data we have developed are not available for comparison. Their narrative conflates the Brazilian lowland with other regions, such as the Llanos de Moxos and other systems in the Bolivian-Peruvian foreland basins, where older archeological sites occur. Their comments about the mineral composition of ADEs appear to contradict recent discoveries (made by some of their co-authors)^12^ which show that some oxides found at our ADE site bear “no relationship to anthropogenic activity” because “their sources are attributed to the weathering of micas, feldspars, mafic minerals (pyroxene), and sodic plagioclase” that are not found locally. To explain the inconsistency between those findings and the current theory of ADE formation, Macedo et al. argue that “sediment depositions in floodplain soils” that “are not related to human occupation” should be considered. That suggestion is consistent with our data which indicate deposition of exogenous materials to the site prior to the invention of agriculture in central Amazonia.

Our study area is on a Tertiary terrace, and we acknowledge in our paper that it lies above the modern 100-year flood height for Manaus. However, significant Pleistocene and Holocene tectonic activity and river aggradation/degradation demonstrably affected the flood height over time. A complex neotectonic history has affected terrace elevations, nutrient deposition, and remobilisation, as well as flood heights and aggradation, resulting in higher base levels that were many metres above flood waters today in past millennia^13–15^. In addition, rivers transported and dispersed sediments from the Andes to the lowland, which were re-mobilised, and re-deposited in patchy patterns, from floodplains several times between 20 and 5 thousand years ago^16–18^. Such mineral inputs by past avulsion events may have occurred earlier in the Quaternary and remain as a relict soil where it has not subsequently eroded^19^. The older weathered sediments on the upper terraces lining the river look nothing like recent alluvium and the distribution of elements and their assemblages at our site are consistent with alluvial deposits in other sites. This process is explained in studies cited by Lombardo et al. (e.g., Pupim et al.), which note several periods of river aggradation, that support our hypothesis.

As explained in our original paper, our data do not preclude a more recent human effect on the local landscape. The wisdom of indigenous populations, manifested in the application of waste materials to agricultural sites (since at least the late Holocene), may have further enriched ADEs or countered their natural degradation. Recent studies^12, 16, 17^, which post-date the studies that Lombardo et al. cite to argue against a geogenic influence, reveal a dynamic neotectonic history and support our hypothesis. Thus, the extent to which other ADE sites originated from depositional processes should be investigated based on evidence that goes beyond those presented by Lombardo et al.

## Future directions

Lombardo et al. compare our hypothesis with others proposed in the 1970s, which disregarded social and ecological science. We find this to be a mischaracterisation of our study. Our data and interpretation of results take into consideration the regional history of land use as well as the local anthropological context of the study site. Indeed, our paper describes evidence of human presence, including a range of diets and amounts of waste reported for pre-Columbian populations. In addition to elemental stocks, the ADEs studied at our site have different particle size distributions, stoichiometric, and isotopic ratios than the surrounding area. Unlike what is proposed by Lombardo et al., those differences cannot be explained by foraging habits. We found signatures of exogenous inputs that are larger than expected from human activity and that do not match the currently accepted chronology of occupation. Thus, as explained in our original paper, an anthropogenic origin for ADEs might be possible, but implausible, because it would require a revision of the history of land use in central Amazonia.

Much remains unknown about the origin of putative ADE sites, about the socioecological history of Amazonia, and about the Quaternary geomorphic history of its rivers. Lombardo et al. defend a decades-old theory without engaging new evidence. They argue that a revision of the current paradigm is not necessary, and in doing so, they imply that we know all that there is to be known about the origin of ADEs. To resolve the matter, our hypothesis should be tested in other sites through interdisciplinary research that combines indigenous knowledge, fluvial geomorphology, and a modern understanding of elemental cycling. Such an approach could uncover the mystery of ADE formation and guide the sustainable use of tropical landscapes going forward.

## Data Availability

The data used to produce all figures present here are available at 10.7264/9qdm-en61
